# A Role for the Nucleosome Assembly Proteins TAF-Iβ and NAP1 in the Activation of BZLF1 Expression and Epstein-Barr Virus Reactivation

**DOI:** 10.1371/journal.pone.0063802

**Published:** 2013-05-14

**Authors:** Sheila Mansouri, Shan Wang, Lori Frappier

**Affiliations:** Department of Molecular Genetics, University of Toronto, Toronto, Ontario, Canada; University of Regensburg, Germany

## Abstract

The reactivation of Epstein-Barr virus (EBV) from latent to lytic infection begins with the expression of the viral BZLF1 gene, leading to a subsequent cascade of viral gene expression and amplification of the EBV genome. Using RNA interference, we show that nucleosome assembly proteins NAP1 and TAF-I positively contribute to EBV reactivation in epithelial cells through the induction of BZLF1 expression. In addition, overexpression of NAP1 or the β isoform of TAF-I (TAF-Iβ) in AGS cells latently infected with EBV was sufficient to induce BZLF1 expression. Chromatin immunoprecipitation experiments performed in AGS-EBV cells showed that TAF-I associated with the BZLF1 promoter upon lytic induction and affected local histone modifications by increasing H3K4 dimethylation and H4K8 acetylation. MLL1, the host protein known to dimethylate H3K4, was found to associate with the BZLF1 promoter upon lytic induction in a TAF-I-dependent manner, and MLL1 depletion decreased BZLF1 expression, confirming its contribution to lytic reactivation. The results indicate that TAF-Iβ promotes BZLF1 expression and subsequent lytic infection by affecting chromatin at the BZLF1 promoter.

## Introduction

Epstein–Barr virus (EBV) is a ubiquitous human gammaherpesvirus that is associated with several human malignancies including B-cell lymphomas, nasopharyngeal carcinoma and gastric carcinoma. EBV is usually found in a latent mode of infection in which a small subset of viral genes are expressed, and EBV genomes persist as multicopy episomes that replicate once per cell cycle [1], [2]. Latent EBV can reactivate to the lytic cycle, which involves expression of over 80 genes, and viral genome amplification to generate linear genomes for packaging in infectious virions.

The switch from latent to lytic infection is triggered by the EBV immediate-early (IE) transcription factor BZLF1 (also called Z, Zta, and ZEBRA). BZLF1 binds to specific DNA sequences to transactivate its own promoter, as well as the promoter of the BRLF1 transcription factor [3], [4], [5], [6]. BZLF1 and BRLF1 then function synergistically to activate the sequential expression of the cascade of EBV lytic genes [7]. BZLF1 also directly contributes to viral replication by binding the origin of lytic DNA replication (oriLyt) and recruiting viral replication proteins [8], [9], [10]. Due to the importance of BZLF1 in EBV reactivation, mechanisms by which its promoter are activated is an active area of study. The cellular transcriptional factors Sp1 and Sp3, phosphorylated c-Jun and CCAAT/enhancer binding protein (C/EBP), have all been shown to associate with the BZLF1 promoter region and contribute to its activation, while ZEB1 binding to BZLF1 promoter elements suppresses BZLF1 expression [3], [11], [12], [13], [14].

EBV episomes in latent infection are complexed with nucleosomes with similar spacing to cellular DNA [15], [16], so it is not surprising that viral gene expression is highly regulated by host proteins that affect chromatin structure. Indeed, the chromatin state of the BZLF1 promoter is an important determinant of BZLF1 expression, and subsequent lytic infection, as this promoter can be activated by histone deacetylase inhibitors such as trichostatin A (TSA) [17], [18], [19]. In addition, three histone chaperone proteins have been shown to contribute to viral transcriptional activation by the EBV EBNA1 protein; bromodomain-containing protein 4 (Brd4), nucleosome assembly protein 1 (NAP1) and template-activating factor Iβ (TAF-Iβ) [20], [21]. All three of these proteins can interact with EBNA1 through one of the two transcriptional activation regions and are recruited by EBNA1 to the viral enhancer element, FR [20], [21], [22]. In addition, EBNA1 recruits TAF-Iβ (also called SET) to the DS origin element of *oriP*, which negatively regulates replication [20]. TAF-Iβ was originally identified, along with its alternatively spliced form TAF-Iα, as a host factors that stimulated DNA replication and transcription of adenovirus core particles *in vitro* [23], [24]. Subsequently, TAF-Iα and β were found to be components of the inhibitor of histone acetylation (INHAT) complex and therefore inhibit p300-mediated protein acetylation [25], [26]. Conversely, TAF-Iβ was also shown to bind p300 and CBP and to stimulate acetylation by CBP [27], [28]. Therefore, through opposing effects on histone acetylation, TAF-Iβ can both activate and repress gene expression.

Since chromatin structure is a major factor in controlling EBV reactivation to the lytic cycle, we explored the roles of NAP1 and TAF-I on EBV lytic reactivation in epithelial cells containing latent EBV genomes. We found that both NAP1 and TAF-I contribute to BZLF1 expression leading to subsequent lytic gene expression and viral amplification. TAF-Iβ was also found to associate with the BZLF1 promoter region and to recruit the host histone methyltransferase MLL1 (mixed lineage leukemia protein-1), affecting histone acetylation and dimethylation.

## Results

### NAP1 and TAF-I Positively Contribute to BZLF1 Expression and the EBV Lytic Cycle

**Figure 1:**
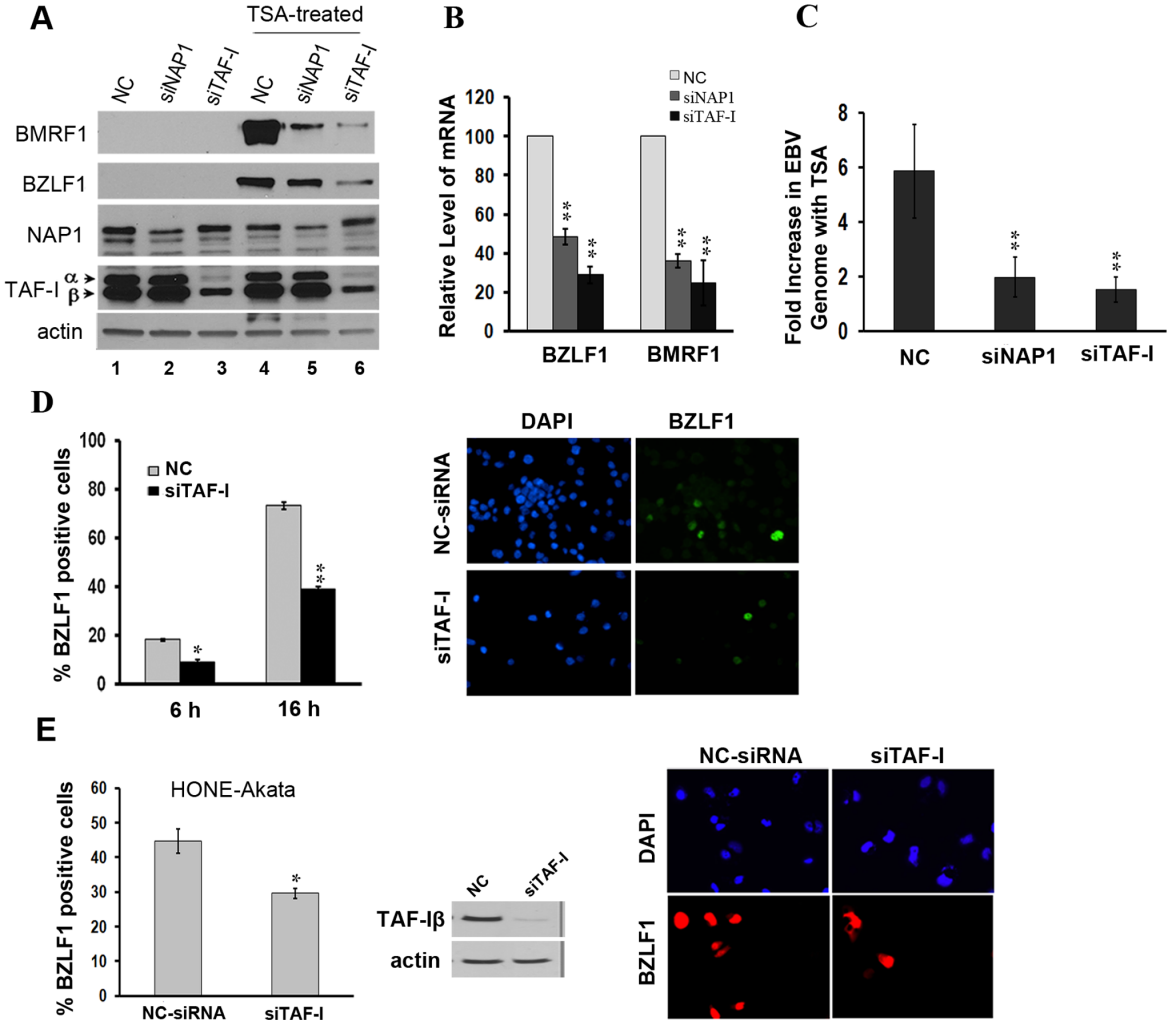
NAP1 and TAF-I contribute to EBV lytic replication. (**A**) AGS-EBV cells were transfected with siRNAs against NAP1, TAF-I, or negative control (NC) siRNA and, 48 hours later, treated with TSA for 24 hours to induce the EBV lytic cycle (lanes 4-6) or left untreated (lanes 1-3). Equal amounts of extracts from these cells were Western blotted using antibodies against NAP1 [52] [46] [45] [45] [45], TAF-I, actin, BZLF1 and BMRF1. (**B**) qPCR analysis of mRNA levels of BZLF1 and BMRF1 in TSA-treated AGS-EBV cells transfected with the indicated siRNAs as in A. Total RNA was isolated and BZLF1 and BMRF1 transcripts were amplified and normalized to GAPDH transcripts. Relative mRNA levels are shown where NC siRNA samples were set to 100. (**C**) EBV episome copy number was determined from samples in B by qPCR of the DS region, which was normalized to cellular GAPDH. The fold increase in EBV genomes after TSA treatment (compared to no TSA treatment) is shown for each siRNA treatment. The data from B and C are from three independent experiments with PCR performed in duplicate for each. (**D**) AGS-EBV cells grown on coverslips were transfected with TAF-I siRNA or NC-siRNA, treated with TSA for 6 or 16 hours, then stained for BZLF1. The percentage of BZLF1-positive cells was counted by immunofluorescence microscopy for 3 independent experiments. A representative image is shown of BZLF1 staining after treatment with TAF-I siRNA or NC-siRNA and 16 hr TSA treatment (all cells were confirmed to be depleted in TAF-I after siTAF-I treatment in an independent experiment). (**E**) HONE1-Akata cells were transfected with siRNA against TAF-I, treated with TSA for 16 hours, then stained for BZLF1. The percentage of BZLF1-positive cells was counted by immunofluorescence microscopy for 3 independent experiments and average values are shown in the bar graph. A Western blot confirming TAF-Iβ silencing and a representative image of the BZLF1 staining are also shown. For all the bar graphs ** = P<0.01, * = 0.01<P<0.05.

To examine the possible roles of NAP1 and TAF-I in EBV reactivation, we down-regulated NAP1 or TAF-I and with siRNA in AGS cells latently infected with EBV (AGS-EBV) and determined the effect on the expression of the BZLF1 lytic switch protein and another lytic protein, BMRF1 (the DNA polymerase processivity factor). Expression of these proteins was examined both before and after induction of the lytic cycle with TSA. As shown in Figure 1A, TSA treatment induced the expression of BZLF1 and BMRF1, however the induction of these proteins was reduced in cells treated with siRNA against NAP1 or TAF-I (targeting both TAF-I and isoforms) as compared to negative control siRNA, even though NAP1 and TAF-I were only partially depleted. TAF-I downregulation in particular dramatically decreased the levels of BZLF1 and BMRF1, perhaps due to more efficient depletion of TAF-I and as compared to NAP1. We also quantified the BZLF1 or BMRF1 transcripts after TSA treatment, by quantitative PCR (qPCR) using primers specific to these mRNAs [29] and normalized these signals to those from cellular GAPDH mRNA (Figure 1B). The mRNA levels of both BZLF1 and BRMF1 were significantly reduced when either NAP1 or TAF-I was depleted relative to the siRNA negative control (P<0.01), and, consistent with the Western blot results, TAF-I depletion had a more pronounced effect. Therefore these results indicate that NAP1 and TAF-I positively contribute to BZLF1 and BMRF1 protein expression by increasing the level of their transcripts.

Since lytic DNA replication leads to amplification of the viral genomes, we also quantified the level of the viral genomes using qPCR as previously described [30] as another measurement of lytic induction. After the siRNA and TSA treatments, total DNA was extracted from AGS-EBV cells and subjected to qPCR using primers specific to the EBV dyad symmetry (DS) element in latent origin of replication, oriP. These values were normalized to those obtained by qPCR using primers for the cellular GAPDH open reading frame. As shown in Figure 1C, TSA treatment increased the EBV genome copy number approximately 6-fold in siRNA negative control cells relative to that present before TSA treatment. This increase represents an average from cells that did and did not respond to the TSA treatment as ∼40% of cells responded by expressing BZLF1 as detected by immunofluorescence microscopy (data not shown). However, when the same experiment was performed in cells depleted for NAP1 or TAF-I, TSA treatment only increased the level of the EBV genomes 2-fold or less, indicating that lytic DNA replication was decreased approximately 3-fold relative to control samples (P<0.01). This provided further verification that NAP1 and TAF-I can positively contribute to EBV lytic reactivation.

Another way to quantify effects on lytic infection is to determine the percentage of cells that express BZLF1. Initially we transfected the AGS-EBV cells with TAF-I siRNA or negative control siRNA then added TSA and imaged the cells for BZLF1 expression by immunofluorescence microscopy 6 or 16 hours post-TSA treatment (Figure 1D; early induction times were necessary to keep cells from detaching from the slides). We focused on TAF-I since NAP1 depletion was inefficient. The results showed TAF-I depletion decreased the percentage of cells entering the lytic cycle approximately 2-fold at both time points, further supporting a positive role for TAF-I in BZLF1 expression. Similarly, TAF-I silencing was also found to decrease the percentage of HONE1-Akata cells (nasopharyngeal carcinoma cells latently infected with EBV) that switched to the lytic cycle upon TSA treatment as determined by BZLF1 expression (Figure 1E).

### Overexpression of TAF-Iβ or NAP1 Results in EBV Reactivation

**Figure 2:**
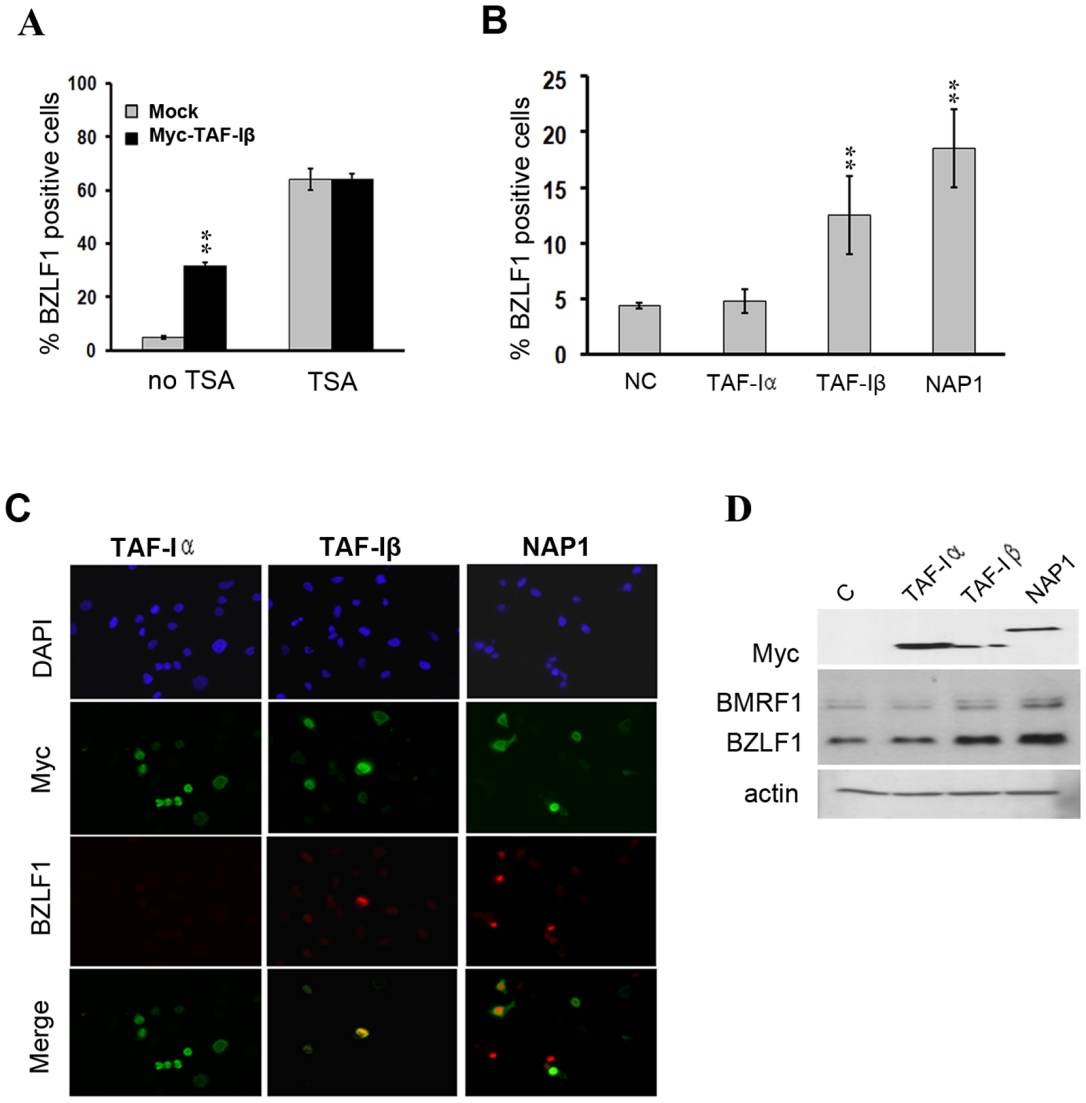
Overexpression of TAF-Iβ or NAP1 induces BZLF1 expression. **(A)** AGS-EBV cells grown on coverslips were transfected with pCMVmyc-TAFIβ, treated with TSA for 16 hours or left untreated then stained with anti-myc and anti-BZLF1 antibodies. The percentage of BZLF1-positive cells was counted separately for myc-positive (myc-TAF-I) and myc-negative (mock) cells on the same slides and average values are plotted from 3 independent experiments. ** = P<0.01. * = 0.01<P<0.05. **(B)** AGS-EBV cells were transfected with pCMVmyc-TAF-Iα, pCMVmyc-TAF-Iβ, pCMVmyc-NAP1 or negative control pCMVmyc and 24 hours later stained with anti-myc and anti-BZLF1 antibodies. The percentage of myc-positive cells that expressed BZLF1 was determined in two independent experiments. (**C**) Representative microscopy images from B. **(D)** A Western blot of the cells in B after transfection with TAF-Iα, TAF-Iβ and NAP1 expression plasmids as compared to mock transfected cells (C).

Since TAF-I and NAP1 appeared to positively contribute to BZLF1 expression, we next tested whether their overexpression was sufficient to induce BZLF1 expression. To this end, we transfected the AGS-EBV cells with a plasmid expressing myc-tagged TAF-Iβ, treated the cells with TSA or left them untreated, then performed immunofluorescence microscopy using antibodies against the myc tag and BZLF1. Since about 30% of the cells expressed myc-TAF-Iβ, we could determine the percentage of myc-positive and myc-negative cells that were BZLF1-positive on the same slides. Remarkably, we found that TAF-I overexpression (in the absence of TSA treatment) was sufficient to induce ∼30% of the cells to express BZLF1; a 7-fold increase over the ∼4% of cells that naturally express BZLF1 (Figure 2A). In contrast, when lytic infection was efficiently induced by TSA treatment (16 hour induction), overexpression of TAF-Iβ did not further increase the frequency of BZLF1 expression (Figure 2A). Together the results provide strong evidence that TAF-I positively contributes to BZLF1 expression.

Our TAF-I silencing experiments do not distinguish between effects of TAF-Iα or TAF-Iβ as both are down-regulated by the siRNA. Therefore we repeated the above overexpression experiments (in the absence of TSA treatment) to determine if myc-TAF-Iα expression was also sufficient to induce BZLF1 expression in AGS-EBV cells. In this set of experiments we also examined the effect of overexpression of myc-NAP1 and another myc-tagged protein domain as a negative control (USP7 catalytic domain; NC in Figure 2B). Quantification of the percentage of BLZF1-expressing cells showed that, like TAF-Iβ, NAP1 overexpression can induce BZLF1 expression, whereas TAF-Iα overexpression did not induce BZLF1 expression, despite being expressed at a higher level than TAF-Iβ (Figure 2B–D). Increased BZLF1 and BMRF1 expression after transfection of the myc-TAF-Iβ or myc-NAP1 construct could also be detected by Western blotting even over the background of ∼70% untransfected cells (Figure 2D). This confirms a positive role for NAP1 and TAF-I in BZLF1 expression in epithelial cells and indicates that the positive role of TAF-I in the lytic switch is most likely due to the action of the TAF-Iβ isoform.

### TAF-I Associates with the BZLF1 Promoter and Affects Histone Modifications

**Figure 3:**
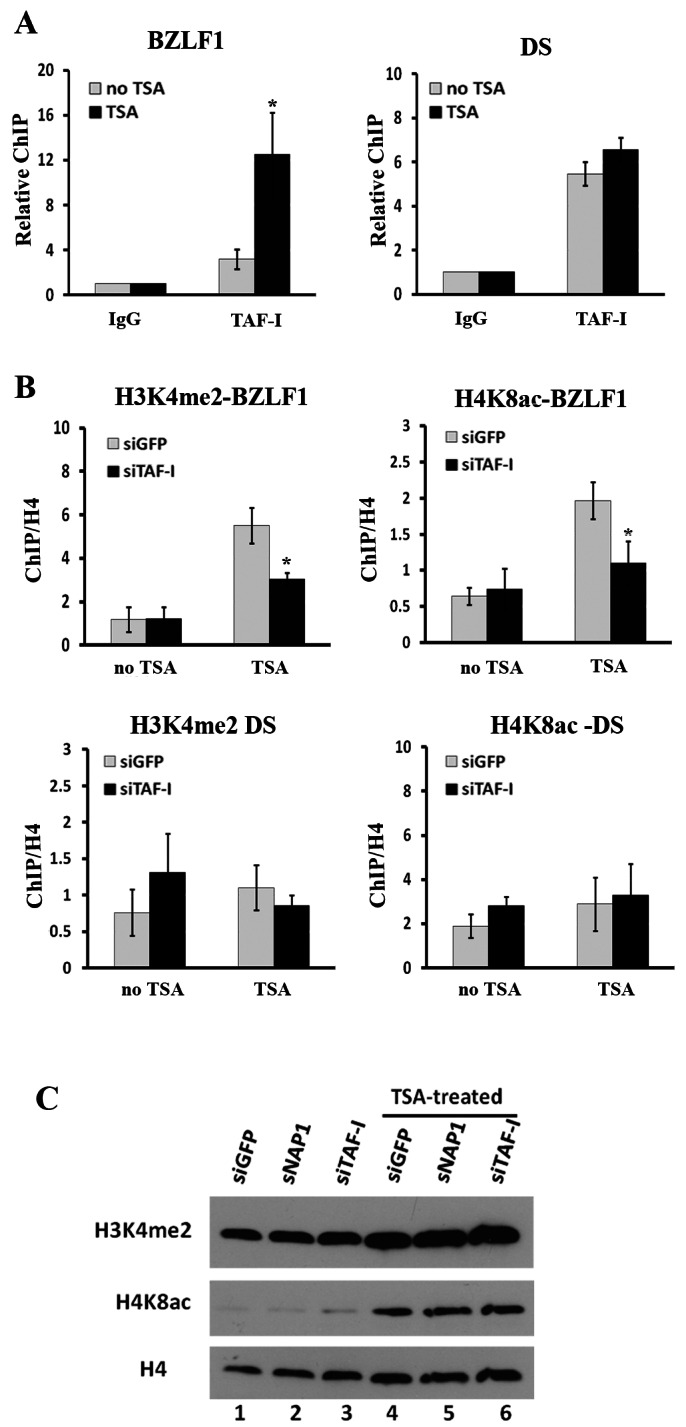
TAF-I binds to the BZLF1 promoter upon EBV lytic reactivation and affects histone modifications. (**A**) ChIP assays were performed on AGS-EBV cells before (grey bars) or after (black bars) TSA treatment using antibodies against TAF-I or nonspecific rabbit IgG. Recovered DNA fragments were quantified by qPCR, using primer sets specific to the DS or the BZLF1 promoter regions as indicated and normalized to signals from total EBV DNA. The signals from the TAF-I samples are shown relative to those from the nonspecific IgG samples, which were set to 1. (**B**) AGS-EBV cells were transfected with siRNAs against TAF-I (black bars) or negative control siRNA against GFP (grey bars), and then treated with TSA for 24 hours or left untreated as indicated. ChIP assays were performed as in A using antibodies against H3K4me2 (left panels), H4K8ac (right panels), or total H4. The amplified signals from the promoter region or DS element were normalized to those from total histone H4. Data is shown from 3 independent experiments with PCR performed in duplicate for each. ** = P<0.01. * = 0.01<P<0.05. (**C**) Equal amounts of total cell lysates from AGS-EBV cells treated with siRNA against TAF-I, NAP1 or GFP with and without TSA treatment were analyzed by Western blotting with the indicated antibodies.

We next asked whether the positive contribution of TAF-Iβ to BZLF1 expression was due to TAF-Iβ acting directly at the BZLF1 promoter. To this end, we performed chromatin immunoprecipitation (ChIP) assays in AGS-EBV cells before and after TSA treatment. The available NAP1 antibodies did not reliably recover NAP1 from the AGS-EBV cells in the ChIP assays, and therefore we focused our ChIP experiments on TAF-Iβ. Chromatin was isolated from AGS-EBV cells with or without TSA treatment, sheared by sonication and subjected to immunoprecipitation with antibodies against TAF-I or control rabbit IgG as previously described [20]. Precipitated DNA was used as a template for qPCR amplification with primers specific for regions of the BZLF1 promoter [29] or the DS element [31] and recovery was normalized to the input EBV DNA to control for increased recovery due to higher levels of the EBV DNA after lytic infection. As shown in Figure 3A, in the absence of TSA (grey bars), the TAF-I antibody (which recognizes both TAF-Iα and TAF-Iβ) recovered both BZLF1 and DS DNA fragments to some degree but resulted in better recovery of the DS, consistent with our previous findings that TAF-I preferentially associates with the DS during latency [20]. However, after TSA treatment (Figure 3A, black bars), the association of TAF-I with the BZLF1 promoter region was significantly increased compared to non-TSA treated samples (4-fold on average; 0.01<P<0.05), whereas no change was observed for TAF-I binding to the DS element. We conclude that there is increased association of TAF-I with the BZLF1 promoter once the lytic cycle has been activated. This was not true for the two other lytic promoters that we examined (for BRLF1 and BMRF1), which were recovered in similar amounts before and after TSA treatment in TAF-I ChIP experiments (Figure S1).

Our finding that TAF-I was associated with the BZLF1 promoter after TSA treatment, coupled with the fact that TAF-I can affect histone modifications [25], [26], [27], [28], prompted us to investigate whether TAF-I affected histone modifications at this promoter. Elevated levels of K8 acetylation on histone H4 (H4K8ac) and K4 dimethylation on histone H3 (H3K4me2) have been reported at the BZLF1 promoter treated with HDAC inhibitors, including TSA, suggesting that increased levels of these histone modifications are important for activation of the BZLF1 promoter [17]. Therefore, we performed ChIP assays with antibodies against H3K4me2, H4K8ac and total H4 to determine if these histone modifications were affected by TAF-I depletion (Figure 3B). To this end, AGS-EBV cells transfected with siRNAs were incubated with or without TSA and then harvested for ChIP assays. As expected, in the siRNA negative control cells, the TSA treatment enhanced the levels of both H3K4me2 and H4K8ac at the BZLF1 promoter (5- and 3-fold, respectively over no TSA treatment; grey bars in left panels) but did not alter these levels at the DS. However, downregulation of TAF-I followed by TSA treatment resulted in reduced levels of H3K4me2 and H4K8ac at the BZLF1 promoter (∼60% of that seen without TAF-I depletion, 0.01<P<0.05) without affecting these levels at the DS. We also performed Western blots on cell extracts with the above treatments to determine if TAF-I depletion globally affected H3K4me2 and H4K8ac levels (Figure 3C). As expected, TSA treatment markedly increased H4K8ac relative to total H4 levels and also increased the amount of H3K4me2, however neither TAF-I nor NAP1 depletion globally affected the level of these histone modifications (compare lanes 5 and 6 to lane 4). This is the expected result since TAF-I and NAP1 should only influence histone modifications near the DNA sites where they are associated, due to recruitment of histone modifying enzymes to this site. The results support a role for TAF-I in activating the BZLF1 promoter by increasing H3K4me2 and H4K8ac in the region of this promoter upon EBV lytic reactivation.

### TAF-I Recruits MLL1 to the BZLF1 Promoter Region

**Figure 4:**
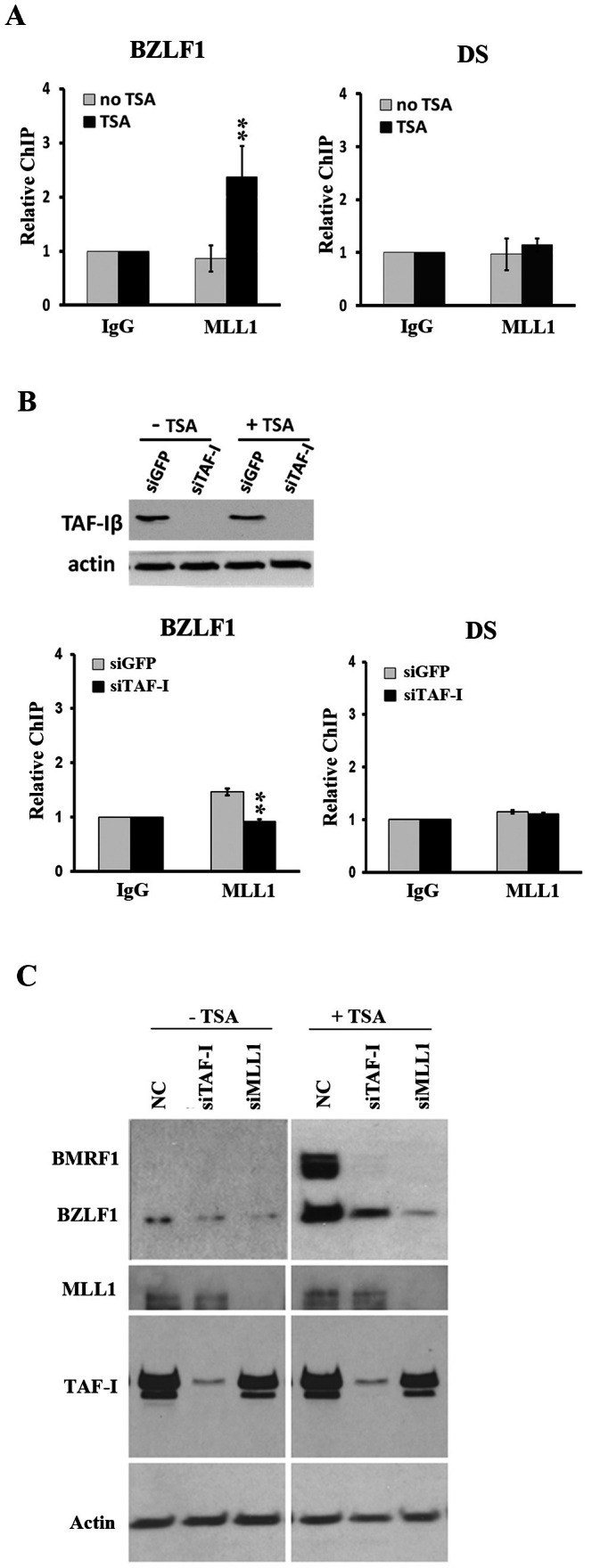
TAF-I recruits MLL1 to the BZLF1 promoter. (**A**) ChIP assays were performed on AGS-EBV cells with (black bars) or without (grey bars) TSA treatment using antibody against MLL1 or nonspecific IgG and primers to amplify the DS or BZLF1 promoter region as indicated. The amplified signals from the ChIP samples were normalized to those from total EBV DNA and shown relative to the nonspecific IgG negative control. (**B**) ChIP assays were performed with MLL1 antibody on TSA-treated AGS-EBV cells transfected with siRNA against TAF-I (black bars) or GFP (grey bars). A Western blot is also shown confirming TAF-I depletion. All ChIP data is from 3 independent experiments with PCR performed in duplicate. ** = P<0.01. **(C)** AGS-EBV cells were transfected with siRNA against TAF-I, MLL1 or negative control siRNA (NC), then treated with TSA (+TSA) or left untreated (-TSA). Western blots were then performed on equal amounts of cell lysates using the indicated antibodies. The image for the BZLF1/BMRF1 blot with –TSA samples was developed using a longer exposure time than that of the +TSA samples in order to detect the low level of spontaneous BZLF1 expression.

TAF-Iβ was previously shown to interact with the MLL1 histone methyltransferase that specifically dimethylates H3K4 [32], [33], [34] and to synergistically upregulate MLL1-mediated transcription [35], [36]. Therefore we examined whether TAF-I might recruit MLL1 to the BZLF1 promoter to generate H3K4me2. To this end, we first asked if MLL1 was associated with the BZLF1 promoter in AGS-EBV cells, by performing ChIP assays using a polyclonal antibody against MLL1. As shown in Figure 4A, MLL1 was not detected at the BZLF1 promoter or the DS element prior to TSA treatment (as compared to the IgG negative control) but was significantly increased at the BZLF1 promoter (but not the DS) upon TSA treatment (P<0.01). Therefore, the association of MLL1 with the BZLF1 promoter region mimicked the behavior of TAF-I. We next asked whether MLL1 binding to the BZLF1 promoter region required TAF-I, by repeating the ChIP assays on TSA-treated AGS-EBV cells transfected with siRNA against TAF-I or negative control siRNA (Figure 4B). TAF-I depletion was found to decrease MLL1 at the BZLF1 promoter (P<0.01), suggesting that MLL1 is recruited to the BZLF1 promoter region in a TAF-I-dependent manner upon activation of the lytic cycle.

To further confirm the role of MLL1 in BZLF1 expression, we silenced MLL1 in AGS-EBV cells and examined the induction of BZLF1 and BMRF1 after TSA treatment (Fig. 4C). MLL1 silencing resulted in a pronounced decrease in the expression of both proteins, confirming its importance in controlling BZLF1 expression. In addition, we examined the effect of MLL1 or TAF-I silencing on the spontaneous BZLF1 expression that occurs in a low percentage of the AGS-EBV cells (in the absence of TSA treatment). Western blots (with longer exposures to be able to detect BZLF1) showed that spontaneous BZLF1 expression was also decreased by TAF-I or MLL1 silencing (Fig. 1C), showing that the contributions of these proteins are not an artifact of TSA treatment.

### EBNA1 Affects TAF-I Association with the BZLF1 Promoter Region

**Figure 5:**
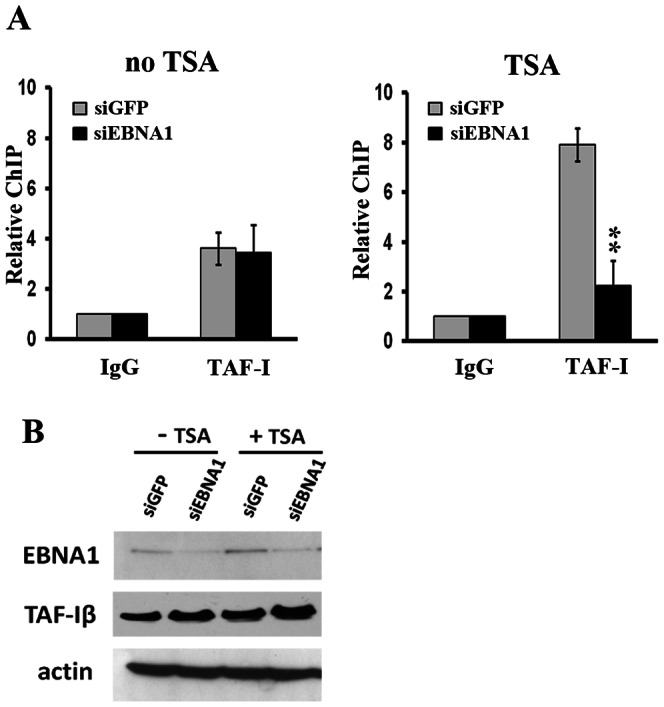
TAF-I binding to the BZLF1 promoter is influenced by EBNA1 upon lytic reactivation. **(A)** ChIP assays for the BZLF1 promoter region were performed on AGS-EBV cells treated with siRNA against EBNA1 (black bars) or GFP (grey bars) before (left graph) or after TSA (right graph) treatment using TAF-I antibody as in Fig. 3A. ** = P<0.01 (**B**) A Western blot showing EBNA1 depletion and TAF-Iβ levels.

Finally, since the EBV EBNA1 protein binds TAF-I [20], [22] and is expressed in both latent and lytic modes of infection, we asked whether the presence of EBNA1 affected TAF-I recruitment to the BZLF1 promoter region. To this end, ChIP assays for TAF-I were repeated in TSA-treated and nontreated AGS-EBV cells with and without EBNA1 depletion as previously described [20]. EBNA1 depletion decreased the level of TAF-I at the BZLF1 promoter after TSA treatment approximately 4-fold (P<0.01), without affecting the lower levels of TAF-I at this region prior to TSA treatment (Figure 5A). However, Western blots of cell lysates showed that total cellular TAF-Iβ levels were not decreased upon EBNA1 depletion (Figure 5B). The results indicate that EBNA1 influences the degree to which TAF-I associates with the BZLF1 promoter region after lytic activation, although we and others have not detected EBNA1 itself at these promoters.

## Discussion

We have identified the related histone chaperones NAP1, and TAF-Iβ as factors contributing to the EBV lytic cycle in epithelial cells through activation of BZLF1 expression. In addition TAF-Iβ was shown to associate with the BZLF1 promoter region to alter histone modifications, including H3K4me2 and H4K8ac, at least in part through recruitment of MLL1. The induction of BZLF1 expression may then be responsible for the downstream effects on other lytic gene expression and DNA replication, although a direct role of TAF-Iβ in regulating other lytic promoters has not been ruled out.

NAP1, TAF-Iα and TAF-Iβ are related proteins that interact with histones through their highly conserved NAP domain [37]. TAF-I exists as both α and β isoforms that dimerize with each other [38], and they were first identified as factors that stimulate the transcription and replication of adenovirus core particles in vitro [23], [24]. More recently they have been shown to affect the transcription of cellular genes through multiple effects on cellular chromatin. In particular, TAF-Iβ can stimulate transcription of specific genes through recruitment of p300/CBP and MLL1, increasing histone acetylation and H3K4 dimethylation [27], [28], [35], [36]. Like TAF-Iβ, NAP1 can stimulate the transcription and replication of adenovirus core particles and activate gene expression through interactions with p300 and CBP [28], [39].

Our data suggests that both TAF-Iβ and NAP1 contribute to the EBV lytic cycle in epithelial cells, at least in part through activation of BZLF1 expression, since the levels of BZLF1 protein and transcripts (after TSA treatment) is decreased by TAF-I or NAP1 downregulation. Furthermore, TAF-Iβ and NAP1 overexpression in the absence of TSA treatment was sufficient to induce BZLF1 expression in a proportion of the latently infected AGS cells. TAF-Iβ was found to associate with the BZLF1 promoter and this association was increased after TSA treatment, suggesting that histone acetylation of the promoter region facilitates TAF-I recruitment. However, the spontaneous BZLF1 expression that occurs in a low percentage of the AGS-EBV cells in the absence of induction is also dependent on TAF-I, showing that the role of TAF-I in BZLF1 expression is not dependent on TSA treatment. The more efficient recruitment of TAF-Iβ to the BZLF1 promoter in a larger percentage of cells after TSA treatment may be the reason that TAF-Iβ overexpression had no additional effect on BZLF1 expression after TSA treatment. In contrast, in the absence of TSA, TAF-Iβ association with the BZLF1 promoter occurs at a small percentage of these promoters and hence may be increased by TAF-I overexpression, leading to the increased expression of BZLF1 that we observed.

TSA treatment increased both H4K8 acetylation and H3K4 dimethylation at the BZLF1 promoter, consistent with the activation of this promoter. However TAF-I depletion decreased both of these histone marks at this promoter after TSA treatment, indicating that these modifications partially depend on TAF-I. Since MLL1 has been shown to dimethylate H3K4 and TAF-Iβ interacts with MLL1, we asked whether TAF-I contributed to the methylation of the BZLF1 promoter region through recruitment of MLL1. Consistent with this hypothesis, like TAF-I, the association of MLL-1 with the BZLF1 promoter region greatly increased after TSA treatment and this association was decreased by TAF-I depletion. Therefore the results are consistent with a model where the association of TAF-I with the BZLF1 promoter alters histone modifications at least in part through recruitment of MLL1. Consistent with this model, MLL1 depletion also decreased BZLF1 expression. Interestingly, MLL1 has also been shown to be important for activation of the immediate early promoters in herpes simplex virus and varicella zoster virus [40], suggesting that it is an important factor for lytic infection by herpes viruses in general.

Finally, we found that depletion of EBNA1, an EBV protein expressed in both latent and lytic EBV infection, greatly decreased the association of TAF-Iβ with the BZLF1 promoter region after TSA treatment. This was not due to a general decrease in TAF-Iβ levels, as EBNA1 depletion did not decrease the cellular levels of TAF-Iβ. Although EBNA1 can directly interact with TAF-Iβ [20], [22], the effect of EBNA1 on the TAF-I-BZLF1 promoter interaction is also unlikely to be due to a role for EBNA1 in mediating the TAF-I-BZLF1 promoter interaction, as we and others have never been able to detect EBNA1 at this promoter. Therefore EBNA1 may indirectly affect the association of TAF-I with the BZLF1 promoter by affecting cellular proteins or processes that lead to alterations in the chromatin structure at the BZLF1 promoter. Consistent with the contribution of EBNA1 to the TAF-I-BZLF1 promoter interaction, EBNA1 was previously shown to increase lytic infection after TSA or TPA treatment [41].

In conclusion, we have identified NAP1 and TAF-Iβ as important factors in EBV lytic infection in epithelial cells. Whether or not NAP1 and TAF-Iβ also contribute to BZLF1 expression and lytic reactivation in B lymphocytes remains to be determined. Although at one time the importance of EBV epithelial infection was questioned [42], the current state of the field strongly supports the view that epithelial cells are an important source of lytic infection in the host [43], [44], [45], [46], and therefore it is important to understand how EBV lytic infection is regulated in epithelial cells. Moreover, such information may be useful to hone strategies to kill EBV-induced epithelial tumours by inducing EBV reactivation [47], [48]. In addition to our studies on EBV reactivation, we have previously shown that NAP1 and TAF-Iβ contribute to EBV latent infection by associating with oriP regions through EBNA1, thereby contributing to EBNA1-mediated transcriptional activation and regulating DNA replication from oriP (TAF-Iβ only) [20]. Therefore these nucleosome assembly proteins play multiple important roles in both phases of EBV infection.

## Materials and Methods

### Cell Culture

AGS-EBV cells are AGS gastric carcinoma cells that have been infected with recombinant EBV in culture [44] and were cultured in RPMI 1640 containing 10% fetal calf serum. HONE-Akata cells were derived from HONE-1 cells by infection with the Akata strain of EBV [49] and maintained in DMEM supplemented with 10% fetal calf serum. Both cell lines were also maintained in G418 (Invitrogen; 400 µg/ml) to select for cells containing recombinant EBV.

### Plasmids and siRNAs

TAF-Iα, TAF-Iβ and NAP1 proteins were expressed in human cells, fused to c-myc, from pCMVmyc using constructs described in Wang et al [20]. The negative control plasmid expressing the myc-tagged USP7 catalytic domain from pCMVmyc is described in Sarkari et al [50]. siRNA targeting NAP1 (GCCGAUAUUUUCCAGUUCUUACAACA) was purchased from Integrated DNA Technologies Inc. siRNAs targeting TAF-I (UCUCUCCAAAGAAUUUCAUCUGAAU), MLL1 (GCUACUGAUCUUGAAUCAATT) [51] or EBNA1 (GGAGGUUCCAACCCGAAAUTT) were synthesized by Invitrogen. siRNA against green fluorescent protein (GFP) (GAACUUCAGGGUCAGCUUGCCG) was used as a negative control as was AllStars siRNA from Qiagen. AGS-EBV or HONE-Akata cells on 10-cm plates were subjected to two rounds of transfection with 100 pmol of siRNA against EBNA1 or NAP1 or 50 pmol of siRNA against TAF-I using Lipofectamine 2000 (Invitrogen), prior to processing as detailed below. For overexpression experiments, AGS-EBV cells in 10-cm plates were transfected with 6 µg of the pCMVmyc expression plasmids using PolyJet (SignaGen) or Lipofectamine 2000.

### Western Blotting and Antibodies

Rabbit polyclonal antibodies to actin (Calbiochem, CP01), BMRF1 (Chemicon MAB8186), TAF-Iβ (Abcam, ab1183), MLL1 (Bethyl Laboratories Inc. A300-086A), myc (SantaCruz sc-70463) and histones H4, H3K4me2, or H4K8ac (Millipore) were used for Western blotting or chromatin immunoprecipitation according to the manufacturers’ protocols. A monoclonal antibody against NAP1 was kindly provided by Dr.Yukio Ishimi and used for Western blotting at a 1∶200 dilution [52]. BZLF1 was detected either with BZLF1 polyclonal antibody (a gift from Dr. Jaap Middeldorp) used at a 1∶1,000 dilution, or with monoclonal antibody from Santa Cruz used at a 1∶5,000 dilution. For Western blotting, antibodies were diluted in blocking buffer containing 5% milk in TBS-T buffer (50 mM Tris [pH 7.5], 150 mM NaCl, 0.1% Tween 20). Blots were washed three times with TBS-T buffer and then incubated with horseradish peroxidase-conjugated secondary antibodies (1∶5,000 dilution, SantaCruz Biotechnology) for 1 hour. Membranes were washed three times with TBS-T, and signals were detected by enhanced chemiluminescence (ECL) assay (Perkin Elmer Life and Analytical Sciences).

### EBV Genome Quantification

Approximately 1×10^6^ AGS-EBV cells treated with various siRNAs and TSA as described above were collected to determine EBV copy number by qPCR as described previously [30], [41]. EBV DNA from each sample was quantified by real-time PCR as described in Sivachandran et al [41] using primers for the DS region of EBV [31] and normalized to the cellular DNA signal at the *GAPDH* locus.

### RNA Isolation and Quantification

Total RNA was isolated from snap-frozen AGS-EBV cell pellets to examine *BZLF1* and *BRMF*1 gene expressions using the TRIzol reagent (Invitrogen). The quantity and quality of the extracted RNA were determined by reading the optical densities at 260 and 280 nm (OD_260/280_) in a NanoDrop spectrophotometer (Thermo Scientific). Total RNA (1 µg) was reverse transcribed in a 25-µl reaction using Transcriptor First Strand cDNA Synthesis Kit (Roche) and random hexamer primers according to the manufacturer’s instructions. Quantitative real-time PCR was performed using 1/4^th^ (for BZLF1 and BMRF1) or 1/20^th^ (for GAPDH) of the cDNA and LightCycle SYBR green I Master mix (Roche) on a Rotorgene qPCR system (Corbett Research). Primers used for mRNA quantification of viral genes *BZLF1* and *BMRF1* [29] and endogenous gene *GAPDH* [53] were as described previously.

### Chromatin Immunoprecipitation (ChIP) Assays

AGS-EBV cells on 10-cm plates were subjected to two rounds of transfection with siRNA against NAP1, TAF-I, EBNA1, or GFP as described above. Forty-eight hours later, cells were treated with 1.5 µM of Trichostatin A (TSA; Cell Signaling Tech.) for 24 hours to induce lytic infection or left untreated. ChIP assays were then performed on cell lysate using antibodies against specific histones, TAF-I or MLL1, or nonspecific IgG as described previously [20]. Recovered DNA fragments were quantified by qPCR using primer sets specific to the DS or BZLF1 promoter region [31] along with SYBR Green qPCR SuperMix (Bio-Rad) in a Rotorgene qPCR system (Corbett Research). Identical qPCR reactions were performed on the cell lysates used for ChIP to provide a measure of the total EBV levels in the sample. Values for DNA fragments recovered with specific antibodies were normalized to the total EBV levels (using the same primer sets) and then shown relative to recovery with nonspecific IgG (to account for background).

### Immunofluorescence Microscopy

Cells grown on coverslips were fixed using 3.4% formaldehyde for 15 min at room temperature and then rinsed briefly in phosphate-buffered saline (PBS). Cells were permeabilized using 0.1% Triton in PBS for 5 min, followed by two 5 min rinses in PBS. Coverslips were blocked for 30 min in 4% bovine serum albumin (BSA) in PBS. Samples were incubated for 1 hour at room temperature with primary antibodies against BZLF1 (1∶300, sc-53904 from Santa Cruz) and/or c-myc (1∶200, sc-789 from Santa Cruz) for detection of myc-tagged TAF-1α, TAF-1β and NAP1. After rinsing in PBS, samples were incubated with the goat anti-mouse Alexa Fluor 488 and/or goat anti-rabbit Alexa Fluor 555 (1∶800; Molecular Probes) secondary antibodies in 4% BSA for 30 min at room temperature. After washing, coverslips were mounted on slides using ProLong Gold antifade medium containing 4′,6-diamidino-2-phenylindole (DAPI) (Invitrogen). Images were obtained using the 40× oil objective on a Leica inverted fluorescence microscope and processed using the OpenLAB (ver.X.0) software program. BZLF1 and myc-positive cells were quantified by counting more than 100 cells per sample, and experiments were performed in triplicate.

## Supporting Information

Figure S1 **TAF-I and MLL1 association with the BRLF1 and BMRF1 promoters is not affected by TSA.** ChIP assays were performed on AGS-EBV cells with (black bars) or without (grey bars) TSA treatment using antibody against TAF-I (A) or MLL1 (B) and primers to amplify the BRLF1 or BMRF1 promoter regions as indicated. The signals from the ChIP samples were normalized to total EBV DNA and value for the “no TSA” sample was set to 1.
(TIF)
