# Supplementary figures and images for: A Role for the Nucleosome Assembly Proteins TAF-Iβ and NAP1 in the Activation of BZLF1 Expression and Epstein-Barr Virus Reactivation
Source: PLoS One. 2013 May 14;8(5):e63802. doi: 10.1371/journal.pone.0063802 (PMC3653829; doi:10.1371/journal.pone.0063802)

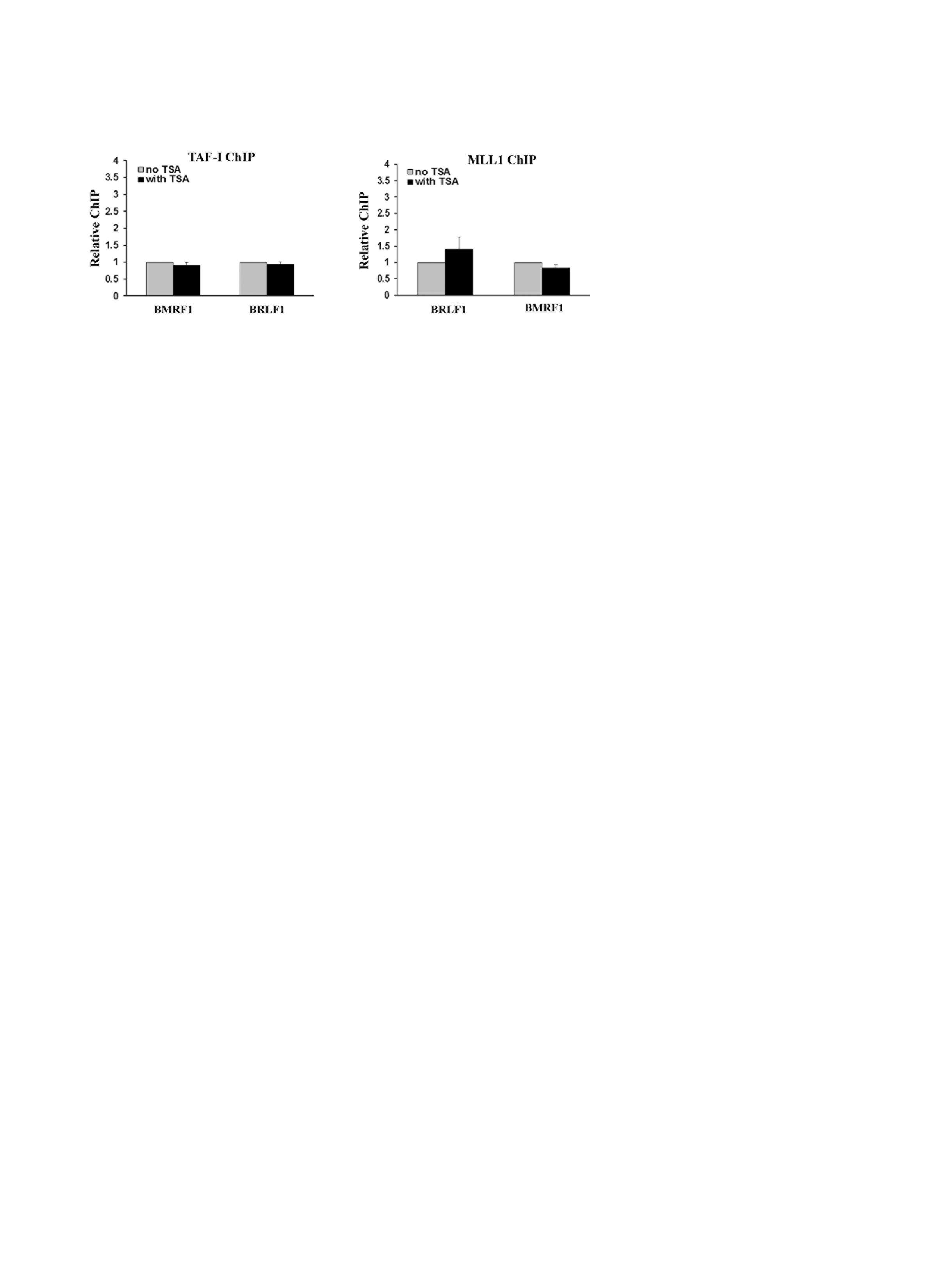

Supplement: Figure S1 — TAF-I and MLL1 association with the BRLF1 and BMRF1 promoters is not affected by TSA. ChIP assays were performed on AGS-EBV cells with (black bars) or without (grey bars) TSA treatment using antibody against TAF-I (A) or MLL1 (B) and primers to amplify the BRLF1 or BMRF1 promoter regions as indicated. The signals from the ChIP samples were normalized to total EBV DNA and value for the “no TSA” sample was set to 1. (TIF) [file pone.0063802.s001.tif]
